# Supplementary material for: Resolving the full spectrum of human genome variation using Linked-Reads
Source: Genome Res. 2019 Apr;29(4):635–45. doi: 10.1101/gr.234443.118 (PMC6442396; doi:10.1101/gr.234443.118)
Supplement: Supplemental Material [file supp_gr.234443.118_Supplemental_longranger-2.2.zip › longranger-2.2.tar.bz2!/lr2.2-public/lib/stan/cmdstan/stan/doc/api/html/base__hmc_8hpp_source.html]

Stan: src/stan/mcmc/hmc/base\_hmc.hpp Source File


|  |  |
| --- | --- |
|  | Stan  2.5.0  probability, sampling & optimization |


- Main Page
- Related Pages
- Namespaces
- Classes
- Files

- File List
- File Members

All Classes Namespaces Files Functions Variables Typedefs Enumerations Enumerator Friends Macros Pages

- src
- stan
- mcmc
- hmc

base\_hmc.hpp

Go to the documentation of this file.

1 #ifndef STAN\_\_MCMC\_\_BASE\_\_HMC\_\_BETA

2 #define STAN\_\_MCMC\_\_BASE\_\_HMC\_\_BETA

3

4 #include <math.h>

5 #include <stdexcept>

6

7 #include <boost/math/special\_functions/fpclassify.hpp>

8 #include <boost/random/variate\_generator.hpp>

9 #include <boost/random/uniform\_01.hpp>

10

11 #include <stan/mcmc/base\_mcmc.hpp>

12 #include <stan/mcmc/hmc/hamiltonians/ps\_point.hpp>

13

14 namespace stan {

15

16  namespace mcmc {

17

18  template <class M, class P, template<class, class> class H,

19  template<class, class> class I, class BaseRNG>

20  class base\_hmc: public base\_mcmc {

21

22  public:

23

24  base\_hmc(M &m, BaseRNG& rng, std::ostream\* o, std::ostream\* e):

25  base\_mcmc(o, e),

26  z\_(m.num\_params\_r()),

27  integrator\_(this->out\_stream\_),

28  hamiltonian\_(m, this->err\_stream\_),

29  rand\_int\_(rng),

30  rand\_uniform\_(rand\_int\_),

31  nom\_epsilon\_(0.1),

32  epsilon\_(nom\_epsilon\_),

33  epsilon\_jitter\_(0.0)

34  {};

35

36  void write\_sampler\_state(std::ostream\* o) {

37  if(!o) return;

38  \*o << "# Step size = " << get\_nominal\_stepsize() << std::endl;

39  z\_.write\_metric(o);

40  }

41

42  void get\_sampler\_diagnostic\_names(std::vector<std::string>& model\_names,

43  std::vector<std::string>& names) {

44  z\_.get\_param\_names(model\_names, names);

45  };

46

47  void get\_sampler\_diagnostics(std::vector<double>& values) {

48  z\_.get\_params(values);

49  };

50

51  void seed(const Eigen::VectorXd& q) {

52  z\_.q = q;

53  }

54

55  void init\_stepsize() {

56

57  ps\_point z\_init(this->z\_);

58

59  this->hamiltonian\_.sample\_p(this->z\_, this->rand\_int\_);

60  this->hamiltonian\_.init(this->z\_);

61

62  double H0 = this->hamiltonian\_.H(this->z\_); // Guaranteed to be finite if randomly initialized

63

64  this->integrator\_.evolve(this->z\_, this->hamiltonian\_, this->nom\_epsilon\_);

65

66  double h = this->hamiltonian\_.H(this->z\_);

67  if (boost::math::isnan(h)) h = std::numeric\_limits<double>::infinity();

68

69  double delta\_H = H0 - h;

70

71  int direction = delta\_H > std::log(0.8) ? 1 : -1;

72

73  while (1) {

74

75  this->z\_.ps\_point::operator=(z\_init);

76

77  this->hamiltonian\_.sample\_p(this->z\_, this->rand\_int\_);

78  this->hamiltonian\_.init(this->z\_);

79

80  double H0 = this->hamiltonian\_.H(this->z\_);

81

82  this->integrator\_.evolve(this->z\_, this->hamiltonian\_, this->nom\_epsilon\_);

83

84  double h = this->hamiltonian\_.H(this->z\_);

85  if (boost::math::isnan(h)) h = std::numeric\_limits<double>::infinity();

86

87  double delta\_H = H0 - h;

88

89  if ((direction == 1) && !(delta\_H > std::log(0.8)))

90  break;

91  else if ((direction == -1) && !(delta\_H < std::log(0.8)))

92  break;

93  else

94  this->nom\_epsilon\_ = ( (direction == 1)

95  ? 2.0 \* this->nom\_epsilon\_

96  : 0.5 \* this->nom\_epsilon\_ );

97

98  if (this->nom\_epsilon\_ > 1e7)

99  throw std::runtime\_error("Posterior is improper. Please check your model.");

100  if (this->nom\_epsilon\_ == 0)

101  throw std::runtime\_error("No acceptably small step size could be found. Perhaps the posterior is not continuous?");

102

103  }

104

105  this->z\_.ps\_point::operator=(z\_init);

106

107  }

108

109  P& z() { return z\_; }

110

111  virtual void set\_nominal\_stepsize(const double e) {

112  if(e > 0) nom\_epsilon\_ = e;

113  }

114

115  double get\_nominal\_stepsize() { return this->nom\_epsilon\_; }

116

117  double get\_current\_stepsize() { return this->epsilon\_; }

118

119  virtual void set\_stepsize\_jitter(const double j) {

120  if(j > 0 && j < 1) epsilon\_jitter\_ = j;

121  }

122

123  double get\_stepsize\_jitter() { return this->epsilon\_jitter\_; }

124

125  void sample\_stepsize() {

126  this->epsilon\_ = this->nom\_epsilon\_;

127  if(this->epsilon\_jitter\_)

128  this->epsilon\_ \*= ( 1.0 + this->epsilon\_jitter\_ \* (2.0 \* this->rand\_uniform\_() - 1.0) );

129  }

130

131  protected:

132

133  P z\_;

134  I<H<M, BaseRNG>, P> integrator\_;

135  H<M, BaseRNG> hamiltonian\_;

136

137  BaseRNG& rand\_int\_;

138

139  // Uniform(0, 1) RNG

140  boost::uniform\_01<BaseRNG&> rand\_uniform\_;

141

142  double nom\_epsilon\_;

143  double epsilon\_;

144  double epsilon\_jitter\_;

145

146  };

147

148  } // mcmc

149

150 } // stan

151

152 #endif

boost::math::isnan

bool isnan(const stan::agrad::var &v)

Checks if the given number is NaN.

**Definition:** boost\_fpclassify.hpp:69

stan::mcmc::base\_hmc::z\_

P z\_

**Definition:** base\_hmc.hpp:133

stan::mcmc::base\_hmc::write\_sampler\_state

void write\_sampler\_state(std::ostream \*o)

**Definition:** base\_hmc.hpp:36

base\_mcmc.hpp

stan::mcmc::base\_hmc::init\_stepsize

void init\_stepsize()

**Definition:** base\_hmc.hpp:55

stan::mcmc::base\_hmc::hamiltonian\_

H< M, BaseRNG > hamiltonian\_

**Definition:** base\_hmc.hpp:135

stan::mcmc::ps\_point

**Definition:** ps\_point.hpp:16

stan::mcmc::base\_hmc::set\_stepsize\_jitter

virtual void set\_stepsize\_jitter(const double j)

**Definition:** base\_hmc.hpp:119

stan::mcmc::base\_hmc::sample\_stepsize

void sample\_stepsize()

**Definition:** base\_hmc.hpp:125

stan::mcmc::base\_hmc::z

P & z()

**Definition:** base\_hmc.hpp:109

stan::mcmc::base\_mcmc::out\_stream\_

std::ostream \* out\_stream\_

**Definition:** base\_mcmc.hpp:44

stan::mcmc::base\_hmc::set\_nominal\_stepsize

virtual void set\_nominal\_stepsize(const double e)

**Definition:** base\_hmc.hpp:111

ps\_point.hpp

stan::mcmc::base\_hmc::nom\_epsilon\_

double nom\_epsilon\_

**Definition:** base\_hmc.hpp:142

stan::mcmc::base\_hmc::rand\_int\_

BaseRNG & rand\_int\_

**Definition:** base\_hmc.hpp:137

stan::mcmc::base\_hmc::epsilon\_jitter\_

double epsilon\_jitter\_

**Definition:** base\_hmc.hpp:144

stan::mcmc::base\_hmc::get\_sampler\_diagnostics

void get\_sampler\_diagnostics(std::vector< double > &values)

**Definition:** base\_hmc.hpp:47

stan::mcmc::base\_hmc

**Definition:** base\_hmc.hpp:20

stan::mcmc::base\_mcmc::err\_stream\_

std::ostream \* err\_stream\_

**Definition:** base\_mcmc.hpp:45

stan::mcmc::base\_hmc::get\_stepsize\_jitter

double get\_stepsize\_jitter()

**Definition:** base\_hmc.hpp:123

stan::math::e

double e()

Return the base of the natural logarithm.

**Definition:** constants.hpp:86

stan::mcmc::base\_hmc::get\_nominal\_stepsize

double get\_nominal\_stepsize()

**Definition:** base\_hmc.hpp:115

stan::mcmc::base\_hmc::get\_sampler\_diagnostic\_names

void get\_sampler\_diagnostic\_names(std::vector< std::string > &model\_names, std::vector< std::string > &names)

**Definition:** base\_hmc.hpp:42

stan::mcmc::base\_hmc::base\_hmc

base\_hmc(M &m, BaseRNG &rng, std::ostream \*o, std::ostream \*e)

**Definition:** base\_hmc.hpp:24

stan::mcmc::base\_hmc::seed

void seed(const Eigen::VectorXd &q)

**Definition:** base\_hmc.hpp:51

stan::mcmc::base\_hmc::rand\_uniform\_

boost::uniform\_01< BaseRNG & > rand\_uniform\_

**Definition:** base\_hmc.hpp:140

stan::agrad::log

fvar< T > log(const fvar< T > &x)

**Definition:** log.hpp:15

stan::mcmc::base\_hmc::get\_current\_stepsize

double get\_current\_stepsize()

**Definition:** base\_hmc.hpp:117

stan::mcmc::base\_hmc::integrator\_

I< H< M, BaseRNG >, P > integrator\_

**Definition:** base\_hmc.hpp:134

stan::mcmc::base\_hmc::epsilon\_

double epsilon\_

**Definition:** base\_hmc.hpp:143

stan::mcmc::base\_mcmc

**Definition:** base\_mcmc.hpp:13


---

[ Stan Home Page ]

*© 2011–2014,
Stan Development Team.*
